# PathDetect-SOM: A Neural Network Approach for the Identification of Pathways in Ligand Binding Simulations

**DOI:** 10.1021/acs.jctc.1c01163

**Published:** 2022-02-25

**Authors:** Stefano Motta, Lara Callea, Laura Bonati, Alessandro Pandini

**Affiliations:** † Department of Earth and Environmental Sciences, University of Milano-Bicocca, Milan 20126, Italy; ‡ Department of Computer Science, Brunel University London, Uxbridge UB8 3PH, U.K.; § The Thomas Young Centre for Theory and Simulation of Materials, London SW7 2AZ, U.K.

## Abstract

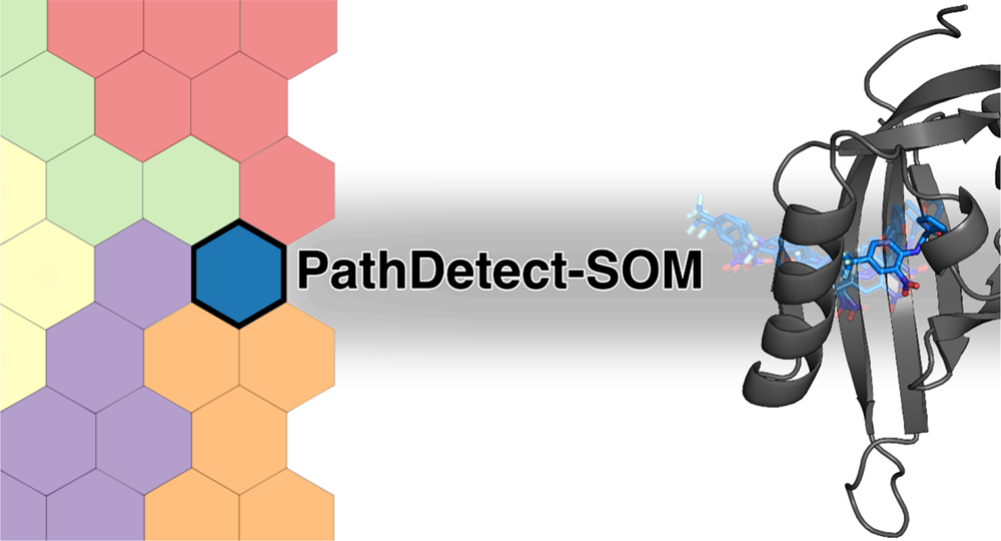

Understanding the process of ligand–protein recognition is important to unveil biological mechanisms and to guide drug discovery and design. Enhanced-sampling molecular dynamics is now routinely used to simulate the ligand binding process, resulting in the need for suitable tools for the analysis of large data sets of binding events. Here, we designed, implemented, and tested PathDetect-SOM, a tool based on self-organizing maps to build concise visual models of the ligand binding pathways sampled along single simulations or replicas. The tool performs a geometric clustering of the trajectories and traces the pathways over an easily interpretable 2D map and, using an approximate transition matrix, it can build a graph model of concurrent pathways. The tool was tested on three study cases representing different types of problems and simulation techniques. A clear reconstruction of the sampled pathways was derived in all cases, and useful information on the energetic features of the processes was recovered. The tool is available at https://github.com/MottaStefano/PathDetect-SOM.

## Introduction

The binding of a ligand to its macromolecular target is a critical event in many cellular processes in living organisms. Understanding ligand–protein recognition and interactions at the molecular level is important to unveil biological mechanisms and to provide the basis for the design and discovery of new drugs.^1,2^

Molecular docking is a well-established computational method to predict the three-dimensional structure and to estimate the binding free energy of a protein–ligand complex.^3,4^ The low computational requirements of this method made it the leading approach for ligand virtual screening. In recent years, due to an impressive increase in computational power, alternative methods based on molecular dynamics (MD) have gained increasing attention for their higher accuracy in modeling ligand–protein binding by considering protein conformational flexibility.^5^ These methods can be classified into two categories: those mainly focused on the bound and unbound states for estimation of the binding free energy and those aimed at reproducing the physical pathway (PP) of binding (and/or unbinding).^6^ Methods that fall in the first category include end-state methods, such as the linear interaction energy (LIE)^7^ and the molecular mechanics Poisson–Boltzmann surface area (MM-PBSA),^8^ and alchemical free-energy perturbation methods, such as thermodynamic integration (TI)^9^ and free-energy perturbation (FEP).^10^

PP methods simulate the complete binding and/or unbinding events, which can in principle lead to the calculation of both thermodynamic and kinetic properties^11^ and to the characterization of relevant states along the pathways. Methods falling within this category include several enhanced-sampling approaches such as steered MD (SMD),^12,13^ metadynamics (MetaD)^14^ and its variations,^15−18^ Gaussian-accelerated MD (GaMD),^19^ scaled MD,^20,21^ τ-RAMD,^22^ MD binding,^23,24^ maze,^25,26^ and CG-MD.^27^ It should be noted that with the increase in computational power due to easier access to high-performance or GPU-based architectures, unbiased simulations are also becoming computationally affordable for the study of long-time-scale processes.^28−31^ The PP methods have the advantage of explicitly simulating key molecular events, such as the protein conformational changes that facilitate ligand access to the binding cavity and the formation of intermediate states. All the above information is fundamental to suggest appropriate modifications of hit compounds in drug-design studies. However, PP methods generally require an extensive sampling of binding/unbinding events to obtain an accurate description of the energy landscape of the process based on reliable statistics. It follows that many events have to be analyzed through several simulation replicas or with a single simulation that describes several re-crossing events. The large amount of data from different replicas or events calls for better automated tools to analyze all the simulated events at once and to provide a clearly interpretable summary picture of the differences in the sampled pathways.

We suggest the use of self-organizing maps (SOMs)^32^ to handle such complex sets of data. An SOM is a type of artificial neural network useful for effective identification of patterns in the data^33−35^ and has been widely used in many fields.^36,37^ The most interesting property of an SOM is that it performs a dimensionality reduction by mapping multidimensional data on the SOM grid, retaining topological relationships between neurons, that is, keeping similar input data close to each other on the map.^33^

Several applications of SOMs to the analysis of biomolecular simulations can be found in the literature,^38−40^ ranging from comparison of the dynamics of different mutants,^41^ clustering of ligand poses in virtual screening,^42^ binding site identification,^43^ identification of blocks for structural alphabets^44−46^ and conformational analysis of loop opening.^47^ More recently, we applied SOMs to the reconstruction of protein unfolding pathways on the basis of several SMD simulation replicas.^48^

Here, we designed, implemented, and tested PathDetect-SOM (pathway detection on SOM), an SOM-based protocol for the analysis of ligand binding/unbinding pathways derived from MD simulations with PP methods. Taking advantage of the properties of SOMs, the tool is able to generate a model that clearly highlights differences in the pathways sampled along a simulation or in different replicas.

The protocol makes it possible to obtain a synthetic view of the sampled conformational space by highlighting the relevant states, to trace the pathways followed by the system on the SOM, and to derive a network model that provides a meaningful representation of the binding/unbinding pathways. We applied this protocol to a range of ligand binding/unbinding simulations with different features, successfully obtaining not only a simple schematic representation of the pathways but also hints about the thermodynamics and/or kinetics of the process. The protocol is implemented as the batch executable R script with a command-line interface that will be accessible to biomolecular practitioners with limited or no familiarity with the R environment.

## Methods

### Overview of the Protocol

**Figure 1:**
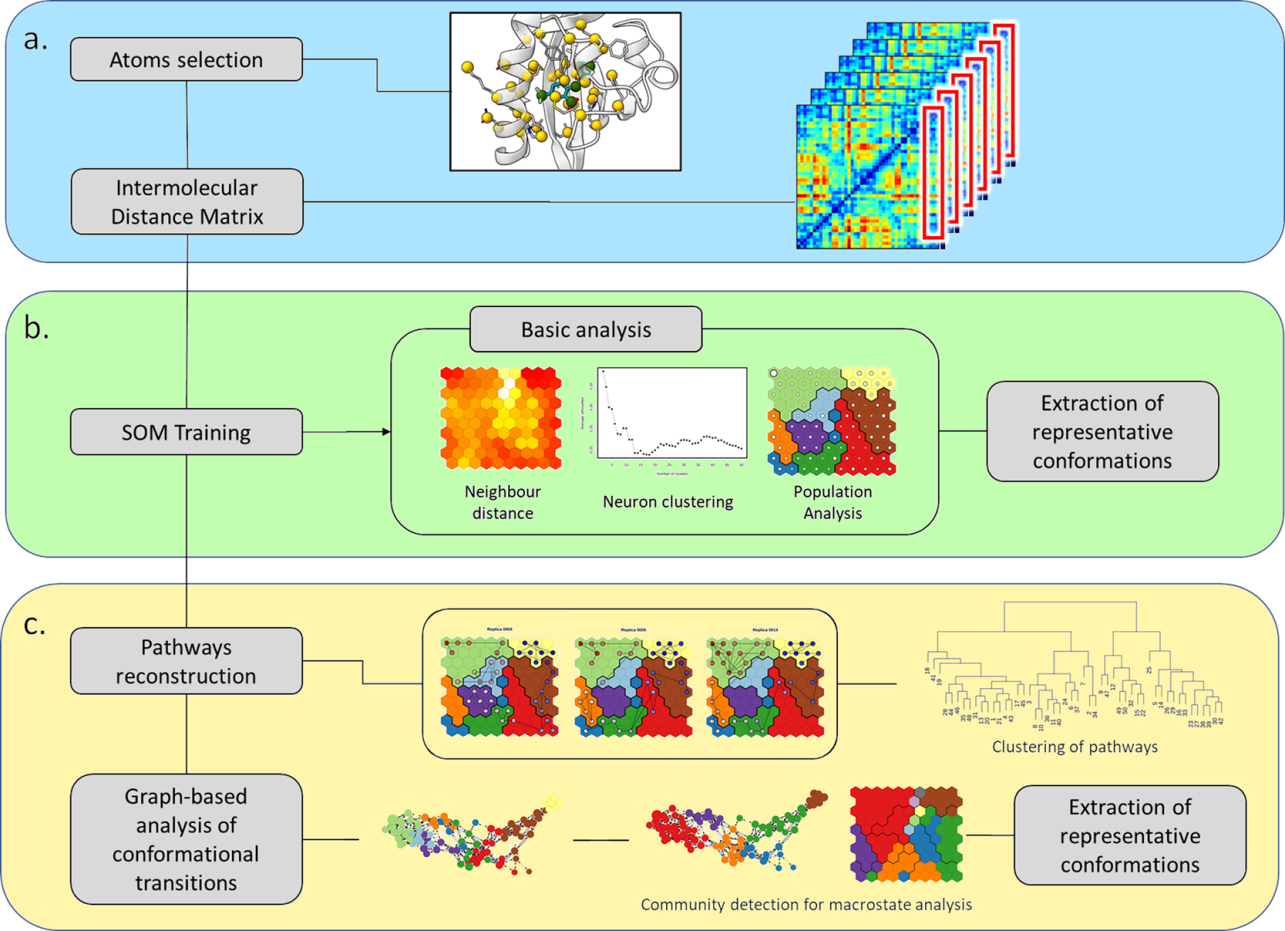
Flowchart of the PathDetect-SOM protocol for ligand binding studies. Data preparation (a); map training and analysis (b); and pathway analysis (c).

PathDetect-SOM is a modular command-line tool based on a three-step protocol (see Figure 1):

(a) The user selects a set of features best describing ligand conformations along the process. If a set of proteins and ligand atoms is provided, the tool will automatically compute the pairwise distances between the protein and ligand sets of atoms. This set of distances will be hereafter referred to as intermolecular distances.

(b) SOM is initialized and trained with the input vectors containing the values of the selected features for all the simulation frames. Each frame is considered as a data point and assigned to the neuron with most similar feature values. During the training process, the feature values of a neuron and its neighbors are adjusted toward the values in the input vector assigned to that neuron. The final prototype vector of each output neuron summarizes the conformations associated with the neuron, and groups of similar conformations are mapped to neighboring neurons. In addition, to offer a more concise picture of the map, after training, the neurons are also grouped to a relatively small number of clusters and the representative conformation of each cluster is saved. Population analysis and average properties can then be visualized on the trained SOM.

(c) The pathways followed during the simulation can be directly traced on the SOM, reconstructing the binding/unbinding pathway. This representation facilitates the identification of regions of the map exclusively sampled by specific simulations. In turn, pathways can be clustered to recover dominant binding events. Finally, a graph-based representation of transitions can be built from the transition matrix calculated at the neuron level. Community detection on this graph can highlight putative macrostates.

PathDetect-SOM is distributed as an R script available under GNU General Public License at https://github.com/MottaStefano/PathDetect-SOM. The repository includes a brief guide and tutorial material based on sample trajectories from the first study case presented in the results.

### Data Preparation

The feature selection is a key step for SOM training. Several features can be used to train the SOM (e.g., the simple cartesian coordinates of a set of atoms, see Supplementary Methods and Figure S1). However, the intermolecular distances are the most suitable choice to accurately describe the ligand–receptor reciprocal orientation. A set of receptor and ligand atoms is chosen for the computation of intermolecular distances. Selected atoms should describe both the binding site and the mouth at the entrance of the binding site. Ideally, both atoms from the backbone and from large or polar/charged side chains should be included when the side chain dynamics and interactions are relevant for binding. Similarly, selected ligand atoms should well describe the core molecular structure and all the relevant lateral groups. The user can provide the filtered trajectory with the coordinates of the chosen atoms in the form of an xvg file, easily obtained using the GROMACS gmx traj command. A capping value is applied to the distances to avoid that training is dominated by information on the unbound states (see Supplementary Methods and Figure S2). Details on the atom selection for the study cases presented here are summarized in Table S1.

### Map Training

The selected features are used to train the SOM using an iterative approach. The map is initialized by assigning random values of the feature vectors to each neuron. In each training cycle, the input vectors representing the single conformations are presented in random order to the map and assigned to the neuron with the closest feature values, also called the best matching unit (BMU). The feature values of the BMU and its neighbors are modified to be closer to the values of the input vector. The magnitude of the modification decreases with the distance from the BMU and along the training. At the end of the iterative process, the resulting SOM preserves the topological relationship between neurons, keeping similar original input data close on the map. In a second step, with the aim of making the map easier to interpret, the neurons are further grouped in a small, but representative, number of clusters by agglomerative hierarchical clustering using Euclidean distances and complete linkage. For each system, the optimal number of clusters can be selected on the basis of silhouette profiles (Figure S3). We propose to choose the number of clusters as the one with the optimal silhouette profile within the 9–15 range. A lower number of clusters would create conformations too coarse for the process that is taking place, while a number higher than 15 would create excessive fragmentation, making the visual interpretation difficult and thus going against the purpose of the tool. A representative structure for each neuron is saved; this is defined as the structure with the feature vector closest to the neuron vector. For each cluster, a representative neuron is also chosen as the one with the feature values closest to the weighted-average feature vector of the neurons belonging to that cluster. In the latter case, the average was performed using the population of each neuron as weight.

In the present work, 10 × 10 sheet-shaped SOMs with a hexagonal lattice shape and without periodic boundary conditions were trained over 5000 training cycles. The neurons were further grouped in a small, but representative, number of clusters, different for each study case, using the cluster analysis approach outlined above.

### Path Analysis

The trained SOM captures the conformational space of several trajectories in a topological map. Therefore, it is possible to reconstruct the path explored by each simulation on the map. Pathways are traced on the SOM based on the annotation of the BMU associated with each frame of the simulation. Given that similar conformations are enforced to be close on the map, the pathways traced on the SOM are usually continuous. Some exceptions to this behavior may arise due to large conformational changes between two consecutive frames in the simulation. Alternatively, discontinuities may highlight important information on the process only visible by projection on the map, that is, the map has the potential to identify and report when partially geometrically similar conformations should be considered dissimilar and separated in the low-dimensional space, probably representative of distinct conformational states. The resulting SOM pathways were also clustered by agglomerative hierarchical clustering using average linkage. Two different distance metrics are implemented in the PathDetect-SOM tool: a time-dependent and a time-independent distance. In the time-dependent version, the distance between the SOM pathways of two simulations is defined as the average distance of the BMUs of each couple of frames. The distance between two BMUs is defined as the Euclidean distance between the position of the neurons on the map. This distance was also used in a previous work by the authors.^48^ In the time-independent version, for each frame of the simulation, the minimum distance between the BMU of the first and the second simulation is computed and averaged over the number of frames. This approach provides a framework to compare simulations evolving at different speeds such as those presented in study case 2. For this type of simulation, indeed, frames to be compared are not at the same position along the replicas due to the different evolution of the simulations. Comparing each frame with the closest frame of the second replica is a time-independent way of performing a distance calculation between two pathways.

An approximate transition matrix between each pair of neurons can be computed from the time-dependent distance approach. The matrix is then transformed into a row stochastic matrix, and a graph is built with nodes representing the neurons and edges with weight proportional to the negative logarithm of the transition probability between the corresponding neurons. Communities of nodes can be detected, and in the present work, we used the walktrap algorithm,^49^ but other methods can be easily applied. A neuron representative of each community is selected as the one with the highest eigenvector centrality score in the subgraph which only contains nodes belonging to the community.

In this work, for the third study case, a commitor analysis was performed using the R library markovchain.^50,51^ This analysis computes the probability of hitting a set of states A before set B starting from different initial states. In this case, the two extremes were the bound and unbound states.

All the analyses were performed in the R statistical environment using the kohonen package^52,53^ for the SOM training and igraph package^54^ for graph construction and analysis.

## Results

The PathDetect-SOM protocol, developed for the analysis of ligand binding/unbinding pathways, is implemented into a command-line tool with the capability to build an SOM representation of the conformations sampled during the MD simulations. Taking advantage of the SOM topological ordering, the tool offers the possibility to visually represent pathways sampled during different events/replicas in a clear 2D representation. Finally, the geometric microstates identified by the SOM (neurons) can be represented as a graph model, built from their transition probabilities. The graph provides a clear representation of the pathways followed during the simulations, facilitating the identification of alternative routes. Community detection on the graph generates a state model analogous to kinetic partitioning.

In the following sections, we present the application of the protocol to three cases that differ for the PP method used to investigate the ligand binding. The study cases were selected to represent PP simulations with different characteristics to highlight the flexibility and general applicability of the PathDetect-SOM tool.

(1) The first case regards a ligand unbinding process studied through several replicas of SMD simulation. The simultaneous evolution of the replicas (due to the constant velocity of the bias) and the use of a directional collective variable (CV) makes this study case simple and optimal for testing some parameters of the tool (tests are discussed in the Supplementary Methods section, Figures S1, S2, S4, and S5 and Tables S2 and S3).

(2) The second study case is a ligand unbinding problem treated with several replicas of infrequent MetaD. This method differs from the SMD used for the first case because the system evolves along the selected CV with a series of small forth and back movements that fill the free-energy basin. As a result, there is no correspondence between the simulation times of different replicas. Moreover, the type of CV chosen in this case is nondirectional and may provide very different unbinding paths.

(3) The third study case consists of a single long MetaD simulation, in which several binding and unbinding events are sampled. In this case, the simulation evolves in all the directions according to two selected CVs, and the ligand has greater freedom than in the previous cases.

All the simulations were performed using GROMACS^55^ patched with PLUMED.^56^

### Ligand Unbinding through Multiple Replicas with Constant Velocity Pulling

The hypoxia inducible factor 2α (HIF-2α) is a pharmacologically relevant transcription factor widely recognized as a target for cancer therapy.^57^ Following the discovery of a buried cavity within the HIF-2α Per-ARNT-SIM-B (PAS-B) domain,^58^ several artificial small molecules were identified as HIF-2α ligands and potential inhibitors of the HIF-2α dimerization with the aryl hydrocarbon receptor nuclear translocator (ARNT).^59−62^ In a recent work, we investigated the unbinding of the THS-020 ligand from the HIF-2α PAS-B domain through SMD simulations.^63^ 50 constant velocity SMD replicas of 25 ns each were used to pull the ligand along the selected CV, namely, the distance between the center of mass of the amino acid atoms lining the cavity and the center of mass of the ligand. The simulations analyzed in this work are those along the preferred entrance to the cavity identified in the previous work (reported as “path 1”).^63^

**Figure 2:**
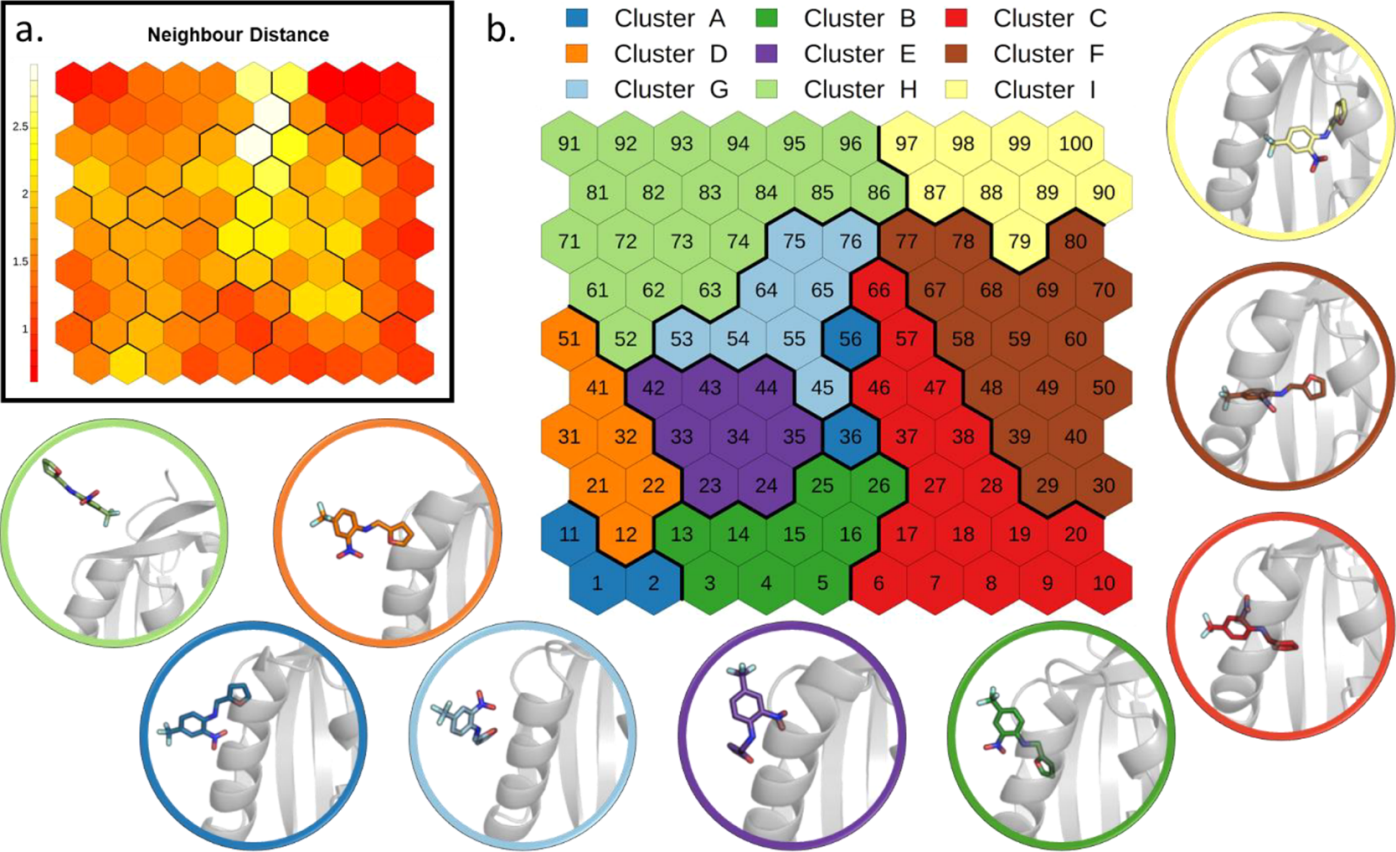
SOM analysis of SMD simulations of THS-020 unbinding from HIF-2α: (a) neighbor distance plot. (b) Clustering of the neurons. The representative conformation of each cluster is depicted in cartoons with the ligand in sticks.

All replicas evolved simultaneously, due to the constant velocity of the bias, and along a directional CV. The trained SOM (Figure 2 and details in the Methods section) shows a distribution of states that ranges from the initial bound state (top-right of the map) to the unbound state (top-left). The neighbor distance plot (Figure 2a) represents the average similarity of a neuron with its neighbors. This map shows a compact group of neurons in correspondence of the bound state and along the right and bottom border of the map. On the contrary, neurons lying at the center of the map display more heterogeneities. In the cluster analysis of SOM neurons (see the Methods section), we identified nine clusters that represent the binding geometries explored by the system following the distance CV used for the SMD simulations (Figure 2b). The representative conformations extracted from the different clusters help to visualize the relevant states sampled.

**Figure 3:**
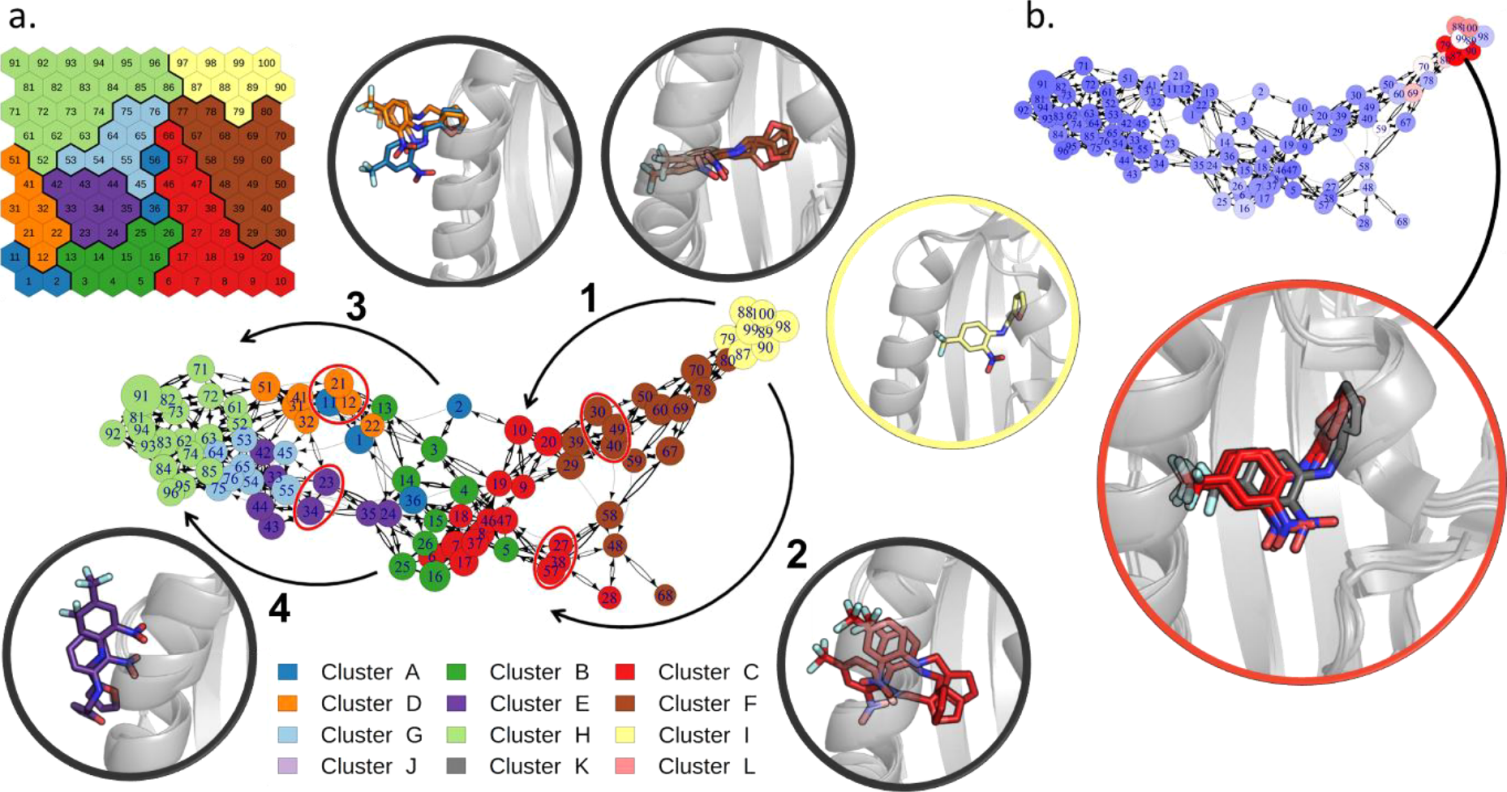
Transition network for the SMD simulations of THS-020 unbinding from HIF-2α. (a) Transition network with its main ramifications explicitly indicated by black arrows (nodes are colored according to the SOM clusters). The representative conformations of neurons that characterize each branch (red circles in the network) and of the bound state (in yellow) are depicted in cartoons with the ligand in sticks. (b) Network colored according to the average SMD force of its frames (from blue to red, increasing values of this property), and the representative conformations of the neurons with the maximum forces, superimposed to the bound state (in gray).

The pathways followed by each replica were then mapped on the SOM (Figure S6). They are quite consistent, since they roughly evolve through the same sequence of clusters, in agreement with the high directionality imposed by the method. However, some recurrent unbinding pathways can be identified with slight differences from each other, as also emerges from the dendrogram in Figure S7. An overview of these pathways is provided by the network graph derived from the transition matrix (see the Methods section), reported in Figure 3a. All the simulations start from the bound state (top-right), in which the ligand presents the nitrobenzene ring parallel to the main helix, with the nitro group pointing toward the lower side of the cavity. Then, some replicas evolve through neurons at the bottom right of the map (branch 1 of the graph), while others follow pathways closer to the center of the map (branch 2 of the graph). While simulations following branch 1, which was sampled in most of the replicas (34 out of 50), show the ligand slightly rotated along its principal axis, those along branch 2 maintain the ligand in an orientation similar to the bound state and rigidly translate it along the pathway. When the nitro group reaches the solvent, however, the two branches merge before a second ramification in the graph appears (branches 3 and 4). Replicas in branch 3 describe a rigid transition of the ligand that maintains the initial bound orientation while those in branch 4 sample conformations with the ligand rotated and bound to the mouth of the cavity. The two final branches appear equally probable (22 replicas though branch 3 and 28 through branch 4).

Finally, we colored neurons according to the average SMD pulling forces applied to the frames belonging to that neuron (Figure 3b). Results show that the pulling of the ligand out of its initial bound state requires the maximum of the force, while the remaining part of the pathway requires less force. We interpreted the peaks of maximum forces as the approximate location of the highest energy barrier to be crossed during unbinding, which corresponds to the energy necessary to pull out the ligand from its initial state.

### Ligand Unbinding through Multiple Replicas with a Bidirectional Sampling

Deoxyhypusine synthase (DHS) is an enzyme responsible for the post-translational hypusination of the eukaryotic initiation factor 5A (eIF5A) that controls cell proliferation and has been linked to cancer.^64^ The involvement in pathogenesis together with the high specificity and functional relevance of the hypusination reaction have made this system an important and promising therapeutic target, stimulating the design and development of inhibitors able to target the hypusination process, including the *N*1-guanyl-1,7-diaminoheptane (GC7). In a recent work by some of the authors, we investigated the unbinding of GC7 from DHS using an approach inspired to infrequent MetaD.^65^ We used the number of contacts between the ligand and the protein binding site atoms as a single CV in 30 replicas of infrequent MetaD that were stopped when the ligand reached an unbound state.

**Figure 4:**
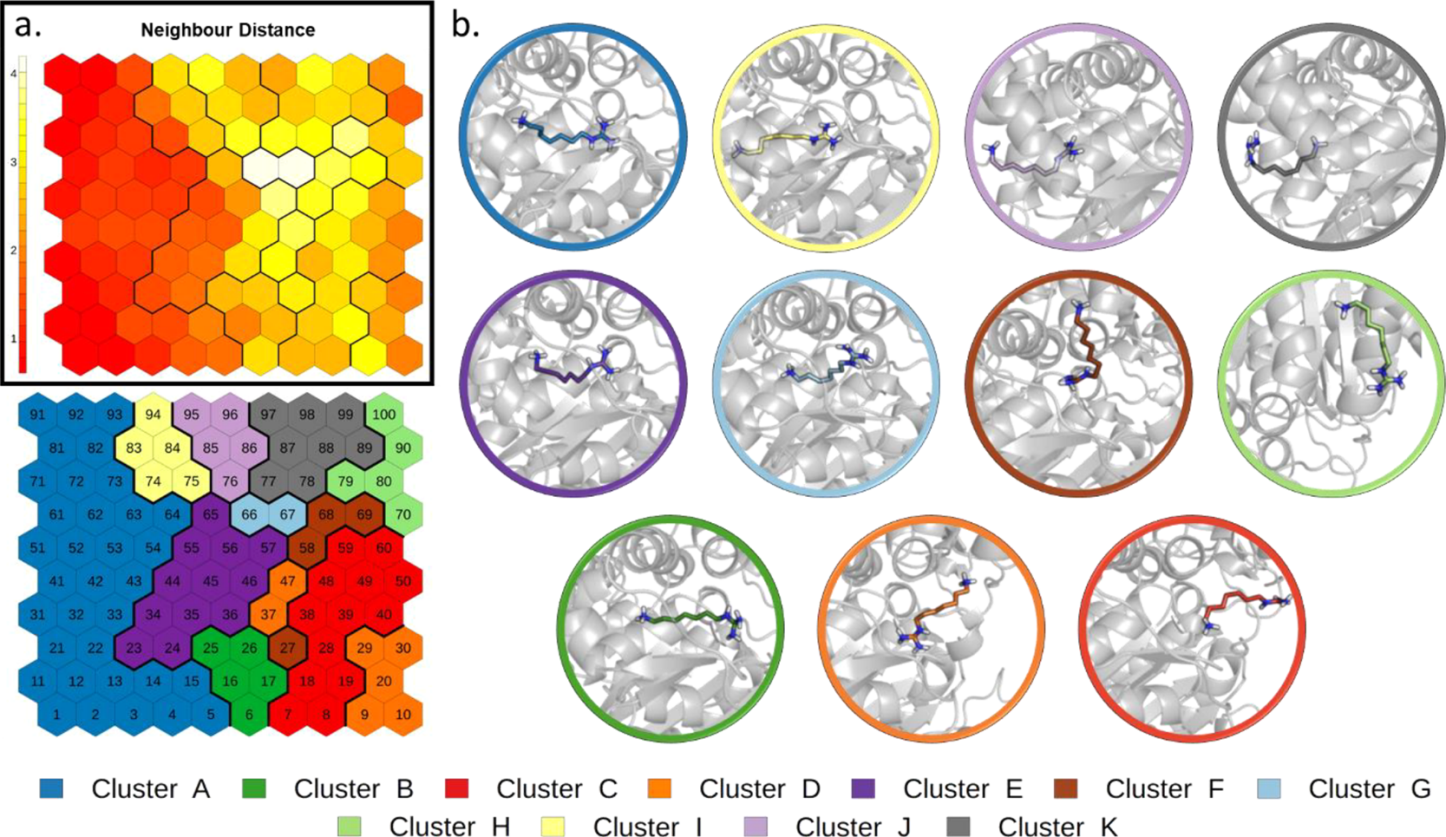
SOM analysis of simulations of GC7 unbinding from DHS. (a) Neighbor distance plot. (b) Clustering of the neurons. The representative conformation of each cluster is depicted in cartoons with the ligand in sticks.

By applying the PathDetect-SOM approach to the above simulations, we obtained the trained SOM shown in Figure 4. The neighbor distance plot (Figure 4a) displays a very compact region on the left side, corresponding to different bound states. All these neurons were grouped together in the neuron clustering phase (cluster A), while the diverse unbound conformations are segregated to the opposite side (Figure 4b).

**Figure 5:**
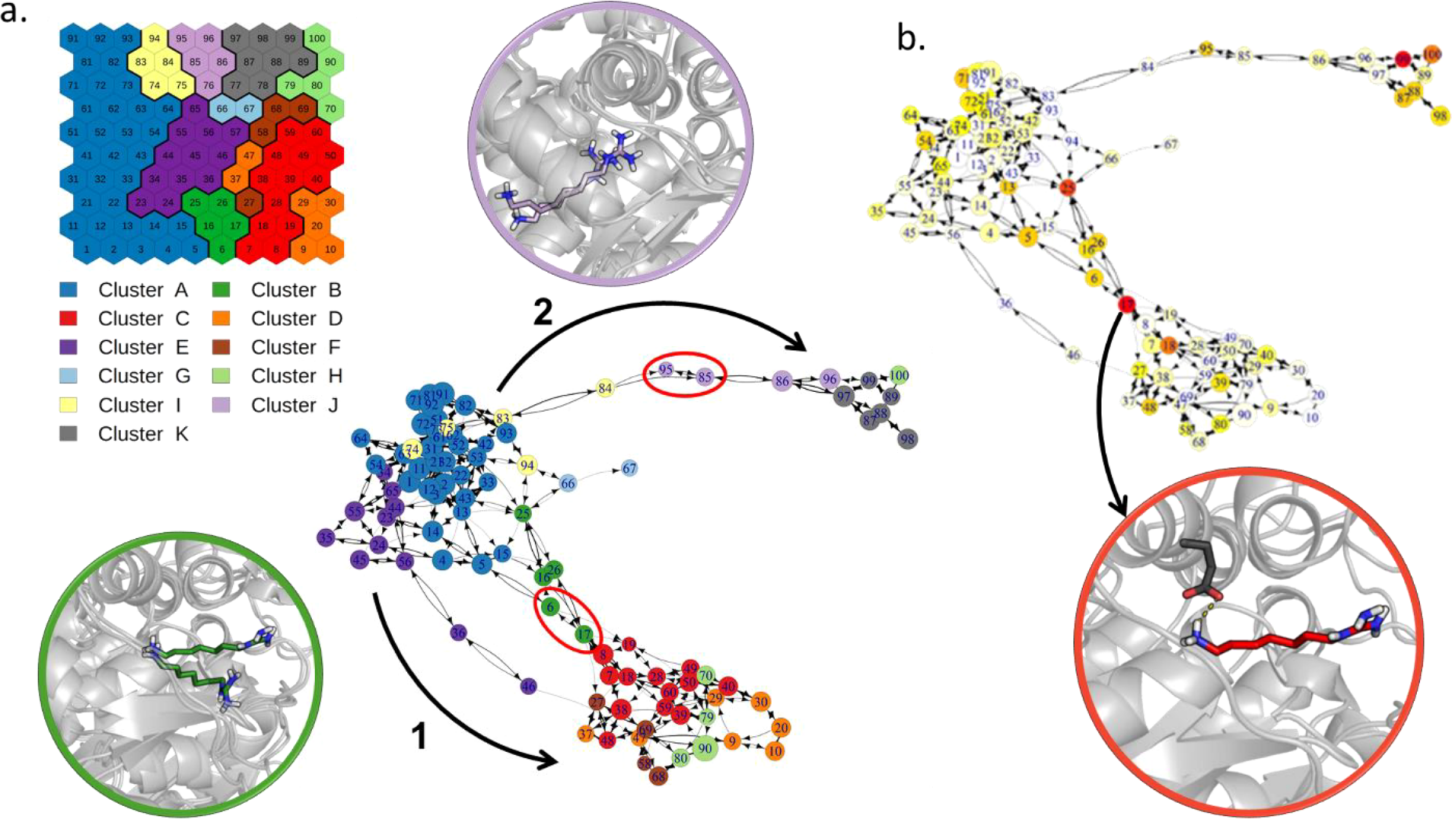
Transition network for the simulations of GC7 unbinding from DHS. (a) Transition network with its ramification explicitly indicated by black arrows (nodes are colored according to the SOM clusters). The representative conformations of neurons that characterize each branch (red circles in the network) are depicted in cartoons with the ligand in sticks. (b) Network colored according to the node betweenness centrality (from white to red, increasing values of this property), and the representative conformations of neuron 17, bottleneck for pathway 1.

Due to the nature of these MetaD simulations, where the system evolves along the CV with a series of small forth and back movements, the direct tracing of the pathways on the map may cause a slight confusion (Figure S8). Moreover, given the lack of correspondence between simulation times of different replicas, we needed to perform a time-independent clustering of pathways (see the Methods section), which allows us to compare replicas of different lengths (dendrogram in Figure S9). Two distinct types of pathways arise from this analysis. Building a network from the transition matrix, as in the previous study case, made the differences between the two pathways more evident (Figure 5a).

The two pathways (branches 1 and 2 of the network, in Figure 5a) lead to different neurons, all describing unbound states. The separation of the unbound states in different neurons is due to the ligand exiting from the two opposite sides of the binding site. Compared to the previous case, this graph is more densely connected due to the bidirectionality of the sampling during the MetaD simulation. Most of the simulations (70%) evolve through branch 1 (Pathway A in the original work^65^) in which the ligand escapes from the side of its guanidine group. The remaining replicas (30%) proceed through an opposite pathway, indicated as branch 2 in the graph, in which the ligand exits from the side of its amino-group (Pathway B in the original work^65^). Interestingly, most of the simulations following branch 1 pass through neuron 17, a node with a high value of betweenness centrality (Figure 5b). As betweenness is calculated as the number of shortest paths through a node,^66^ neuron 17 is a critical conformation to observe the bound/unbound transition. The representative conformation of this neuron shows the characteristic of the intermediate state hypothesized in the previous work,^65^ namely, a stable salt bridge of the ligand primary amine group with Glu137.

### Ligand Binding/Unbinding through a Single Metadynamic Simulation

As a third study case, we applied the PathDetect-SOM protocol to a single MetaD simulation of ligand binding. The system under study is the same presented in Ligand Unbinding through Multiple Replicas with Constant Velocity Pulling: the THS-020 binding to HIF-2α. In a previous work, starting from the SMD simulations, we built a path CV and used well-tempered MetaD to enhance the sampling along the selected CV and to reconstruct the free-energy landscape of the process.^63^ During the 1.8 μs of MetaD simulation, we observed a high number of binding and unbinding events.

**Figure 6:**
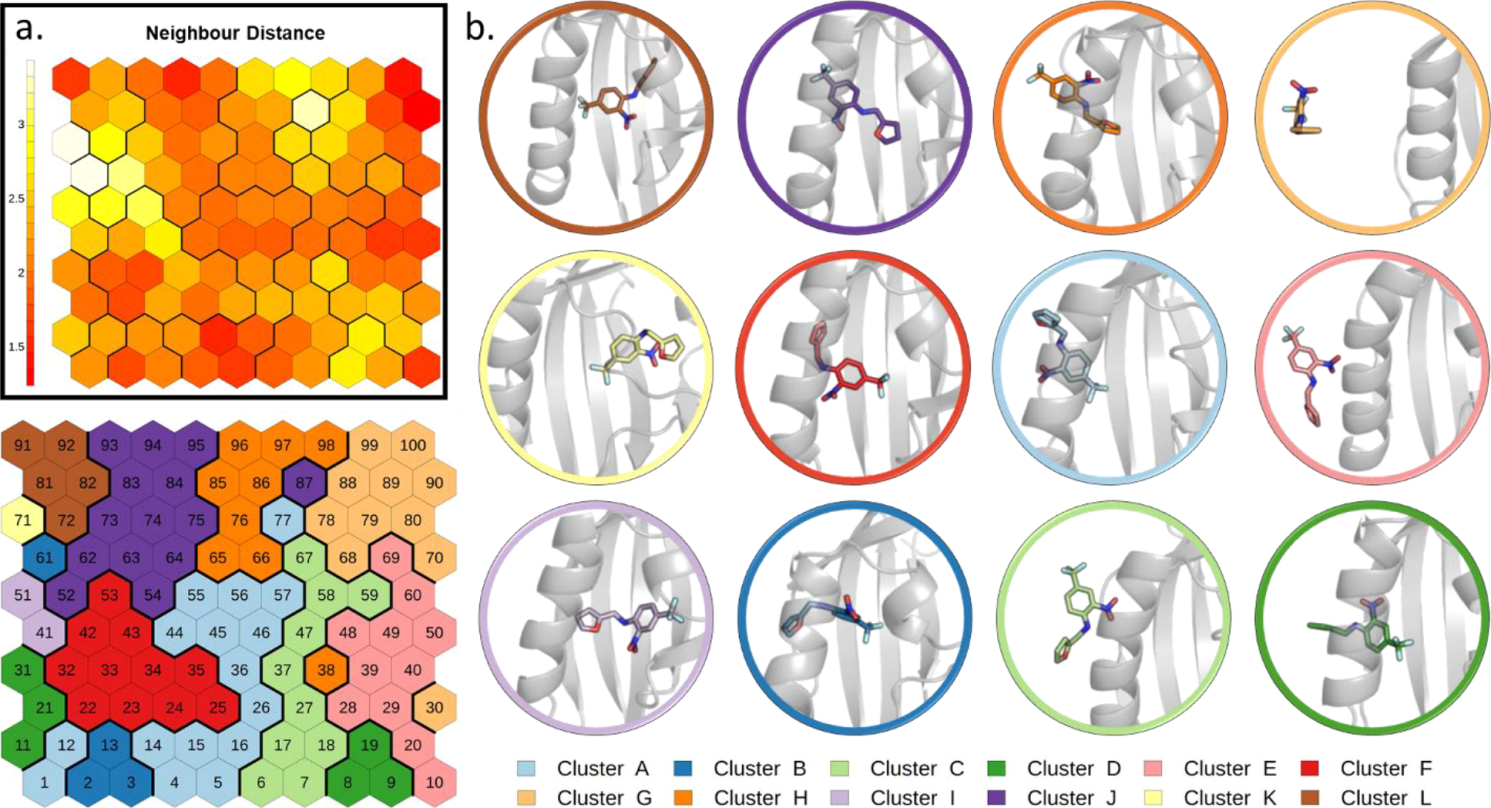
SOM trained with MetaD simulations of THS-020 binding to HIF-2α. (a) Neighbor distance plot. (b) Clustering of the neuron vectors. The representative conformation of each cluster is depicted in cartoons with the ligand in sticks.

The trained SOM (Figure 6 and details in the Methods section) presents the starting bound conformation in the top-left corner (cluster L) and the completely unbound conformation in the top-right corner (cluster G). Due to the conformational freedom along the *z*(*r*) CV of the path CV (which represents the distance from the reference path), the ligand can also rotate and sample alternative bound conformations. This is the case of cluster I, which contains conformations in which the ligand is rotated 180° with respect of the X-ray starting structure.

For the sake of comparison with the free-energy landscape previously identified by the MetaD calculation,^63^ we mapped the frames belonging to each free-energy basin on the SOM (Figure S10). We found that conformations belonging to each of these basins generally map in few close neurons, belonging to the same cluster on the map.

**Figure 7:**
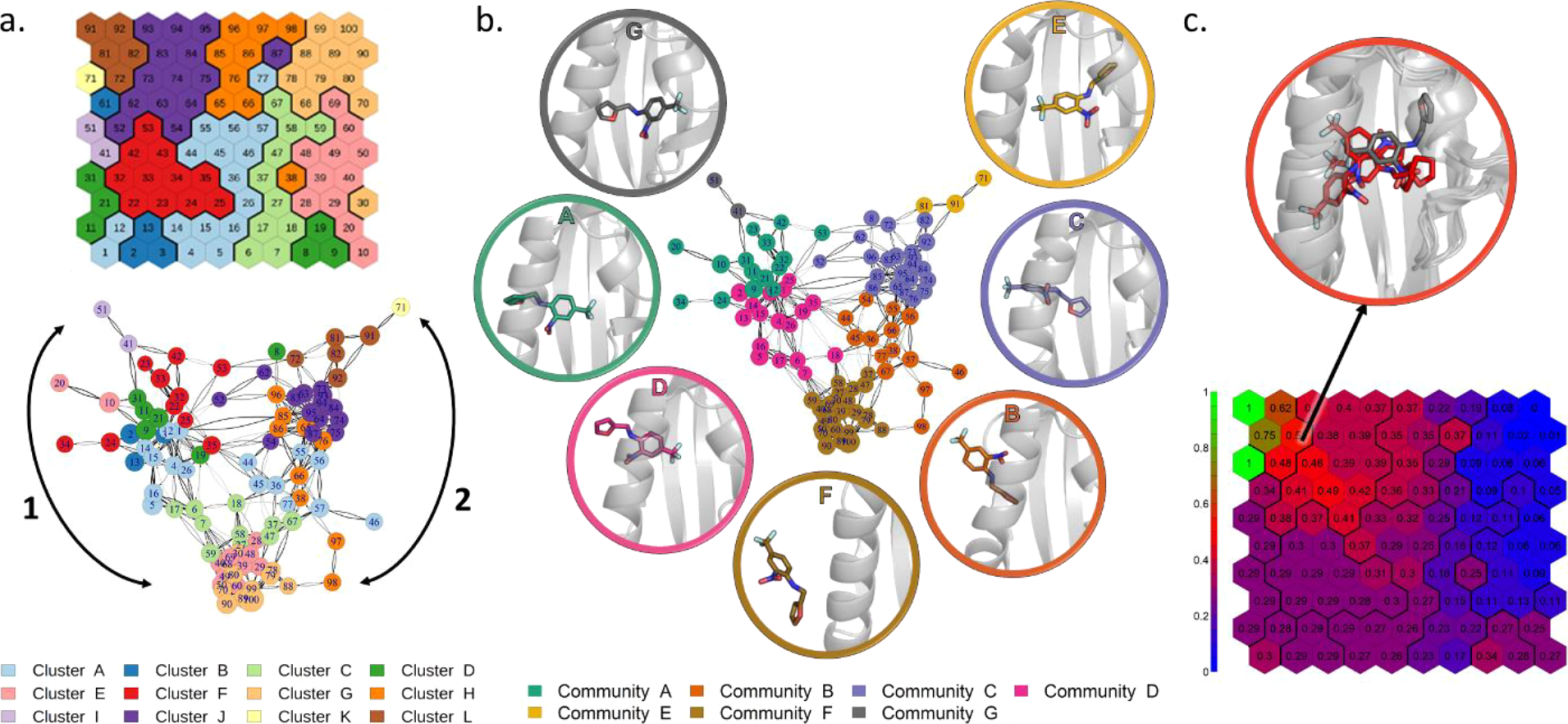
Transition network for the MetaD simulation of THS-020 binding to HIF-2α. (a) Transition network with main pathways indicated by black arrows (nodes are colored according to the SOM clustering). (b) Communities identified by the walktrap method represented on the network (nodes are colored according to the different communities). The representative conformations of the communities are depicted in cartoons with the ligand in sticks. (c) Committor probability analysis. The representative conformations of neurons with a committor probability of about 0.5 are reported in red sticks and X-ray starting conformation in gray sticks.

In this study case, the direct tracing of pathways on the SOM is difficult due to the unique long simulation that samples multiple binding/unbinding events. The set of pathways is better represented on the SOM in the form of a movie (Supplementary Movie 1). However, the transition network analysis proposed in the PathDetect-SOM protocol is capable of providing a clear representation of the pathways sampled during the MetaD simulation (Figure 7a).

As shown in Figure 7a, there are two main branches: branch 1 connects the crystallographic-like bound conformation to the unbound state, while branch 2 follows the unbinding of an alternative binding mode (cluster I). Only a small number of connections between the two branches are present, indicating that the ligand cannot freely rotate within the binding site, and it preferentially unbinds and rebinds to interconvert between the two bound states.

The previous study cases sampled only one unbinding event for each replica and, for this reason, the graph model only describes the interconnection between states along the unbinding pathway. In this last case, due to the MetaD sampling of several binding/unbinding events, the obtained graph takes into account connections along both directions and thus contains more information about the kinetic of the process. Indeed, assuming that the ligand remains trapped for a sufficient time inside an energy minimum, the communities identified with the walktrap method exhibits the properties of kinetic clustering (Figure 7b). It is important to consider that, for an accurate calculation of kinetic properties, the transition matrix should take into account the effect of the bias potential deposited during the simulation. For this reason, this approach can provide quantitative results only if the analyzed simulation is unbiased or if a proper reweighting procedure to the transition matrix is applied. With the aim of showing the potential of the tool, we present the results obtained from the previously published MetaD simulation, without performing any reweighting procedure. The same approach applied to an unbiased simulation, or with a reweight of the transition matrix, would provide accurate description of the kinetic of the process instead of a simple indication of the energy barrier position. The identified communities well represent the ensemble of metastable states sampled along the process. Along both the branches, it is possible to identify a small community for the bound state (communities E and G); a community in which the ligand is still completely inside the binding cavity and did not reach the unbound state (C and A); a community in which the ligand is located at the mouth of the cavity, but it is already partially immersed in the solvent (B and D); and a community for the completely unbound state (F). Moreover, the transitions between communities may be associated with conformational changes with high energy barriers. Focusing on transitions between communities B and C, and between communities A and D, it seems that they are associated with the conformational changes necessary to observe ligand binding. Indeed, nodes at the boundary of these two pairs of communities display higher average RMSD values for residues at the mouth of the cavity involved in the recognition process (Figure S11).

Finally, we performed a committor analysis: we computed the probability of ending in the crystallographic-like bound conformation (neuron 91, in community E) before reaching the unbound conformation (neuron 100, community F) starting from each neuron (Figure 7c). Given that the transition state is expected to have an equal chance of going to either states, configurations with a committor of approximately 0.50 can be considered at the transition state. In the present case, the energetic barrier seems to be located around conformations close to neurons 63, 72, 73, and 82 (in community C, Figure 7c). These conformations are located at the boundaries between communities E and C and are near to the bound state, in agreement with the conclusions drawn from the SMD simulations (Ligand Unbinding through Multiple Replicas with Constant Velocity Pulling).

## Discussion

Data from MD simulations can contain extremely useful information on molecular processes, but it does not lead to simple canonical analysis protocols: system-specific and problem-specific strategies are often required to extract information from increasingly large trajectory files. Planning and designing appropriate strategies can be a very difficult task, and it often requires the development of ad hoc scripts for advanced analysis and the use of dedicated analysis tools.

Several general-purpose tools for the analysis of MD trajectories are available, including GROMACS analysis tools,^67^ CPPTRAJ,^68^ VMD,^69^ MDAnalysis,^70^ Bio3D,^71^ and MDTraj.^72^ All these tools provide basic post-processing analysis such as RMSD, RMSF, radius of gyration, hbond, and contact maps. Some of them are built-in tools distributed along with the main simulation engine (GROMACS analysis tools and CPPTRAJ), while others are python or R libraries that provide a flexible framework for complex analysis (MDAnalysis, Bio3D, and MDTraj) but require the user to develop an ad hoc code.

Among the most advanced post-processing methods, Markov state models (MSMs) are often used to develop a complete kinetic model of the process under investigation.^73,74^ These types of analyses are often complex and require a high level of expertise by the user to obtain reliable results. For this reason, they are difficult to implement as an automated user-friendly protocol. Moreover, effective use of MSMs requires that simulated data meet strict sampling conditions, such as a lag time sufficiently long to produce a Markovian-state decomposition.^75^ This implies that this method can only be used when the aggregate simulation time is in the order of hundreds of microseconds or more. Moreover, development of MSMs using enhanced-sampling MD requires reweighting procedures that nowadays are still at an early stage of development.^76^

Here, we presented a tool based on SOMs specifically designed for the analysis of ligand binding pathways sampled in simulations by means of an automated protocol. Our development takes inspiration from other tools based on SOMs already developed by our group and others,^39^ some of which with fast implementation on GPU.^38^ These tools have been successfully applied to MD data, but they were mainly focused on clustering of macromolecular conformations and not on pathway analysis.

The PathDetect-SOM tool does not have any sampling condition and can be applied to MD simulations that sample multiple ligand binding events. While it cannot be directly used to compute stationary quantities and long-time kinetics (unless one demonstrates that the criteria for MSMs are met), it provides an immediate interpretation of the pathways sampled during the simulation and can give hints about the thermodynamics and kinetics of the process. Recently, an analysis of camphor unbinding pathways from cytochrome P450cam has been performed using the t-distributed stochastic neighbor embedding (t-SNE) dimensionality reduction method.^77^ Authors obtained a two-dimensional representation of the ligand trajectories that facilitate interpretation and helped in grouping similar pathways. Here, we take advantage of SOM properties to obtain a similar dimensionality reduction, with the intrinsic advantage of the resulting segregation of conformations in local microstates (neurons) that immediately allows the building of an approximate transition matrix. Moreover, SOM was also demonstrated as a powerful tool for the comparison of simulations performed with different simulation parameters.^48^

In this work, we tested the PathDetect-SOM tool on a range of ligand binding/unbinding simulations with different features. In all cases, the pathways were successfully characterized and mapped over an intuitive 2D map, thus confirming the general applicability of the protocol. Moreover, depending on the simulation type, several hints regarding the energetics of the process were obtained. In the first study case, we exploited the possibility of re-mapping a property, the SMD pulling forces, on the SOM neurons in order to identify the location of the highest unbinding energy barrier along the simulation (corresponding to the frames with the largest values of the pulling forces). In the second study case, the transition graph and the betweenness centrality score of the nodes suggested the obligate transition across a neuron for the unbinding across pathway 1. Finally, in the third study case, we computed some interesting properties starting from the approximate transition matrix. Here, a reweighting procedure should have been performed to account for the effect of the bias applied during the simulation. Depending on the type of simulation, different reweighting schemes can be applied. If the bias is time-independent, the most simple and effective reweighting procedures are TRAM^78^ and DHAM.^79^ If the bias is time-dependent, on the other hand, the situation becomes more complicated, and the reweighting procedures that can be applied are limited. For example, a reweighting approach for MetaD simulations based on the Girsanov theorem was recently proposed by the group of B Keller.^80,81^ However, random number and the force at every time step are required to calculate the relative path probability, and for this reason, the reweighting factors are computed on the fly (using a patched MD engine) during the simulation and cannot be derived in a post-processing step. With the aim of showing the potential of the tool, we present the results obtained on a MetaD simulation, without performing any reweighting procedure, but accurate kinetic properties can be derived if one analyzes unbiased simulations, or accurately reweighted trajectories. In the presented study case, the committor analysis suggested the location of the energy barrier on the SOM, while determination of the communities in the transition graph led to the identification of kinetic macrostates. As the above properties were computed from the approximate transition matrix, their accuracy strictly depends on the extension of the sampling.

PathDetect-SOM has been implemented in the form of an R batch script with an easy command-line interface. While the tool was primarily designed for ligand binding studies, it can be applied to many other types of simulations (unfolding, protein–protein, or protein–peptide binding) by appropriate choice arguments on the command-line input. The batch script format offers easiness of use with flexibility of customization through simple command-line options. As future development, the tool can be extended and included in an R package to offer expert users the possibility to develop ad hoc extensions to the analyses. The tool is open source and freely available with a brief guide and tutorials at https://github.com/MottaStefano/PathDetect-SOM.

## Supporting Information Available

Selection of optimal parameter values for SOM training; details for feature calculations of each system; comparison of dendrograms by the Cophenetic correlation matrix calculation; comparison of dendrograms by the Baker correlation matrix calculation; SOM trained using RMSD; SOMs trained with different capping values for the distances; Silhouette profiles for the three study cases; SOM trained using the periodic boundary condition; dendrograms of hierarchical clustering of the pathways for study case 1 obtained using different SOM dimensions; pathways of different replicas traced on SOM for study case 1; dendrograms of hierarchical clustering of the pathways for study case 1; pathways of different replicas traced on SOM for study case 2; dendrograms of hierarchical clustering of the pathways for study case 2; mapping of frames belonging to each free-energy basin on the SOM for study case 3; and transition network for study case 3, colored according to protein conformational change (PDF)
The set of pathways represented on the SOM (MP4)

## Abbreviations

MD: molecular dynamics
PP: physical pathway
LIE: linear interaction energy
MM-PBSA: molecular mechanics Poisson–Boltzmann surface area
TI: thermodynamic integration
SMD: steered molecular dynamics
MetaD: metadynamics
GaMD: Gaussian-accelerated molecular dynamics
SOM: self-organizing map
PathDetect-SOM: pathway detection on SOM
dRMSD: distance root mean square deviation
RMSD: root mean square deviation
BMU: best matching unit
CV: collective variable
HIF-2α: hypoxia inducible factor 2α
PAS-B: Per-ARNT-SIM-B
ARNT: aryl hydrocarbon receptor nuclear translocator
DHS: deoxyhypusine synthase
eIF5A: eukaryotic initiation factor 5A
GC7: *N*1-guanyl-1,7-diaminoheptane
MSM: Markov state model.
