# Supplementary material for: PathDetect-SOM: A Neural Network Approach for the Identification of Pathways in Ligand Binding Simulations
Source: J Chem Theory Comput. 2022 Feb 25;18(3):1957–68. doi: 10.1021/acs.jctc.1c01163 (PMC8908765; doi:10.1021/acs.jctc.1c01163)
Supplement: Supplementary file 1 — ct1c01163_si_001.pdf [file ct1c01163_si_001.pdf]

PathDetect-SOM: A Neural Network Approach for the Identification of Pathways in Ligand Binding Simulations

Stefano Motta[1], Lara Callea[1], Laura Bonati[1], Alessandro Pandini[2,3*]*

1 Department of Earth and Environmental Sciences, University of Milano-Bicocca, Milan, Italy.

2 Department of Computer Science, Brunel University London, Uxbridge, United Kingdom.

3 The Thomas Young Centre for Theory and Simulation of Materials, London, United Kingdom.

SUPPLEMENTARY METHODS

Selection of optimal parameter values for SOM training

The selection of parameters and their values for the training of SOMs was based on preliminary analysis of the SMD simulations of THS-020 unbinding from HIF-2α (the first study-case). Here, details concerning the choice of features describing the conformations and associated distance measures, the choice of a capping value for distances, the type of periodic boundary conditions, and the map size are discussed.

Features: The PathDetect-SOM tool can train the map according to different features representing the input conformations from MD trajectories. As each feature has a specific relevant distance measure, user options are based on the type of distance measure: the RMSD (Root Mean Square Deviation) or the dRMSD (distance RMSD). In the case of the RMSD, the SOM is directly trained with atomic coordinates (in the case under study, the coordinates of the heavy atoms of the ligand). This means that simulation frames should be pre-aligned (in this case, on the protein Cα atoms). In the case of the dRMSD, the SOM is trained with a set of distances (in this case, the intermolecular distances). Results from each measure are reported in Figure S1. The choice of the RMSD (Figure S1a) generates a large cluster (C) whose neurons describe both bound and pre-bound states. This becomes evident by mapping the protein-ligand contacts on the SOM (contacts were considered when two atoms are closer than 4.0 Å, Figure S1b) and observing that cluster C includes neurons that exhibit both high and low numbers of contacts. When the ligand is outside the cavity (low number of contacts, blue neurons in Figure S1b), the variability of its conformations is so wide that it hides the changes in the ligand-protein distances that occur along the binding pathways. The results shown in the main text were obtained using dRMSD as distance type and generated a more consistent and informative description of the pathways. When studying a protein-ligand binding process, the intermolecular distances (dRMSD) are the best choice, because the SOM is directly trained on the information relevant for the process as recorded by the changes in intermolecular interactions and, in addition, there is no requirement for preliminary structural superposition.

Capping value: This parameter can be used to set a maximum user-defined value to all the distances between the selected ligand and protein atoms that are greater than this value. SOMs

were trained with different capping values: no capping, 8 Å and 12 Å. To understand how this parameter affects the assignment of simulation frames to the neurons, and consequently the pathway description, the number of ligand-protein contacts was calculated for every frame of the simulation (contacts were considered when two atoms are closer than 4.0 Å, Figure S2). Using the lowest capping value, 8 Å (Figure S2a), the neurons describing the bound state cover most of the map, and only few neurons describe the last portion of the unbinding process. Indeed, a jump in the number of contacts between the neuron containing the unbound state (bottom left corner of the map) and its neighbors is visible. Without the use of capping (Figure S2c), the neurons describing the unbound state cover more than 50% of the map, with poor description of the recognition process. On the contrary, using a value of 12 Å (Figure S2b), a balanced description of all steps of the binding/unbinding process is observed, and the number of contacts gradually changes across the neurons. Given that the map is sensitive to the number of distances reaching the capping value when the ligand gets in the unbound state, it is advisable to adjust the capping value based on the length of the binding pathway within the cavity. A good starting value for the capping could be the distance between residues lying at the bottom and at the mouth of the cavity.

Periodic boundary conditions: Another parameter that can be chosen by the user is the periodicity of the SOM. If the SOM grid is periodic across the boundaries, the neurons at the right boundary will be neighbors of neurons at the left boundary, as well as those on the top and bottom boundaries. The SOM was trained with and without periodic boundary conditions. The main difference is evident when pathways are traced on the SOM. The binding pathways cross the boundary when training is done with periodic boundary conditions (Figure S4) leading to difficulties in interpreting the time evolution of the process. In addition, the neuron clusters

become fragmented across the map, making it difficult to interpret the distribution of the different conformational states in the clusters. The results shown in the main text were obtained without periodic boundary conditions; this choice led to consistent pathways more clearly traceable on the SOM and to easier identification of the different states across the neuron clustering.

Map size: Finally, a relevant parameter for SOM training is its dimension. With the aim of investigating the effect of different map size on pathways clustering, SOMs were trained using different map sizes (8x8, 10x10, 12x12, 16x16, and 20x20). Dendrograms of hierarchical clustering of the pathways obtained for each size of the map (Figure S5) were compared by calculating both the Cophenetic coefficient[1] and Baker[2] Gamma Index. Through the two coefficients, it is possible to evaluate how statistically similar two or more Hierarchical clustering trees are. Specifically, the first one is a measure of how faithfully a dendrogram preserves pairwise distances between the original data points; while the second one is a measure of association, and it is defined as the rank correlation between the stages at which pairs of objects combine in each of the different trees. The values can range between -1 to 1; with near 0 values meaning that the trees are not statistically similar. The analysis was performed using the dendextend R package[3]. Tables S1 and S2, reported the pairwise comparison between maps with different size. Maps with size in the range of 8x8 to 16x16 present a high degree of similarity (values generally between 0.8 and 0.9), which means that there were only very small effects on pathways clustering. When the map size is increased above 16x16, the similarity decreases, thus the differences in the pathways clustering become slightly higher. This could be due to an excessive fragmentation of the map. Moreover, this choice has an impact on the quality of the neuron transition matrix, as increasing the map size creates smaller and more accurate microstates but reduce the number of observed

transitions. Here we proposed a balanced choice (10x10) that could be modulated based on the total number of frames of the simulations, the number of observed transitions and the variety of states sampled during the simulations.

Table S1: Details for feature calculations of each system

System	Atom selection:	Capping value (nm)
HIF-2α – THS020	S246 - OG; H248 - NE2; H248 – CA;<br>M252 – CE; M252 - CA; F254 - CZ;<br>F254 - CA; A277 - CB; F280 - CA;<br>Y281 - OH; Y281 - CA; N288 - CG;<br>N288 - CA; M289 - CE; M289 - CA;<br>K291 - NZ; K291 - CA; S292 - OG;<br>H293 - NE2; H293 - CA; N295 - CG;<br>N295 - CA; L296 - CG; L296 - CA;<br>V302 - CB; V303 - CA; S304 - OG;<br>G305 - CA; Q306 - CA; Y307 - OH;<br>Y307 - CA; M309 - CE; M309 - CA;<br>T321 - OG1; T321 - CA; I337 - CB;<br>C339 - SG; C339 - CA; N341 - CG;<br>N341 - CA	1.2
**System**	**Atom selection:**	**Capping value (nm)**
DHS – GC7	Chain A:<br>K260 - CA; H261 - CA; H261 - NE2;<br>N265 - CA; L268 - CA; L268 – CG;<br>M269 - CA; E284 - CA; E284 – CD;<br>G287 - CA; S288 - CA; D289 - CA;<br>D289 - CG; A292 - CA; E296 – CA;<br>E296 - CD; W300 - CH2; K302 - NZ<br><br>Chain B:<br>N79 - CA; N79 - CG; G106 – CA;<br>E109 - CD; E110 - CD; N137 – CA;<br>R138 - CA; I139 – CA; G140 – CA;<br>Y149 - CA; D211 - CA; S213 – CA;<br>S213 - OG; D216 - CA; D216 - CG	1.6

Table S2: Comparison of dendrograms obtained from different SOM dimensions by Cophenetic correlation matrix calculation.

	8x8	10x10	12x12	16x16	20x20
8x8	1.00	0.84	0.85	0.89	0.74
10x10	0.84	1.00	0.79	0.85	0.74
12x12	0.85	0.79	1.00	0.77	0.67
16x16	0.89	0.85	0.77	1.00	0.79
20x20	0.74	0.74	0.67	0.79	1.00

Table S3: Comparison of dendrograms obtained from different SOM dimensions by Baker correlation matrix calculation.

	8x8	10x10	12x12	16x16	20x20
8x8	1.00	0.86	0.88	0.91	0.73
10x10	0.86	1.00	0.78	0.90	0.75
12x12	0.88	0.78	1.00	0.80	0.65
16x16	0.91	0.90	0.80	1.00	0.79
20x20	0.73	0.75	0.65	0.79	1.00

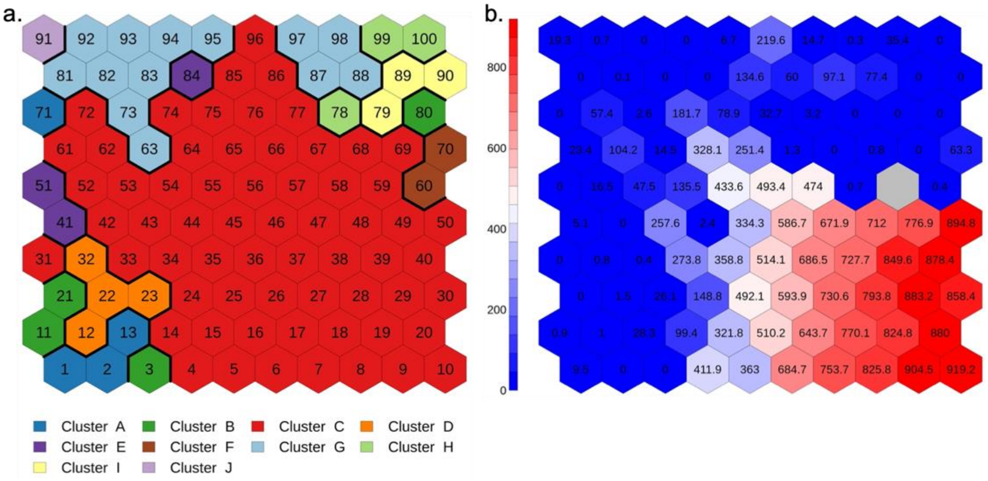

Figure S1: SOM trained using RMSD. (a) clustering of neurons; (b) protein-ligand contacts plotted on SOM. Each neuron is colored according to the average number of contacts in the frames belonging to it. High number of contacts are depicted in red and assigned to neurons describing the bound state.

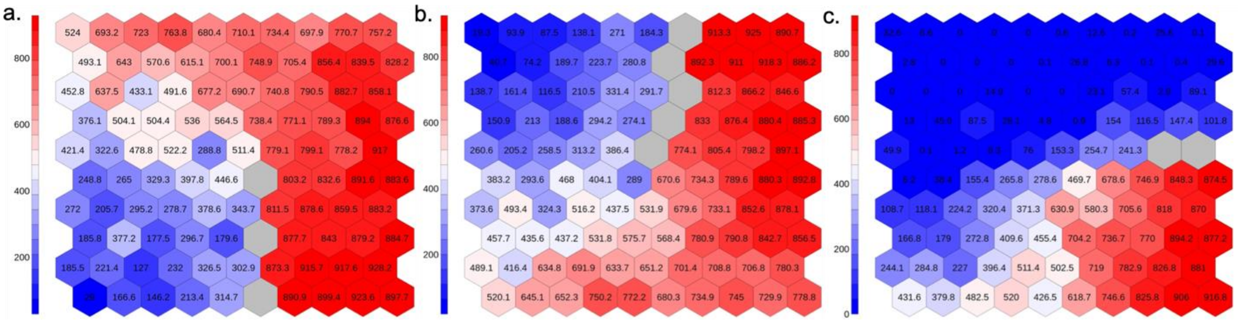

Figure S2: SOMs trained with different capping values for the distances. Number of protein-ligand contacts are plotted on the SOMs: each neuron is colored according to the average number of contacts in the frames belonging to it; high number of contacts are depicted in red and assigned to neurons describing the bound state. (a) SOM trained with 8 Å as capping value, (b) SOM trained with 12 Å as capping value, (c) SOM trained without capping value.

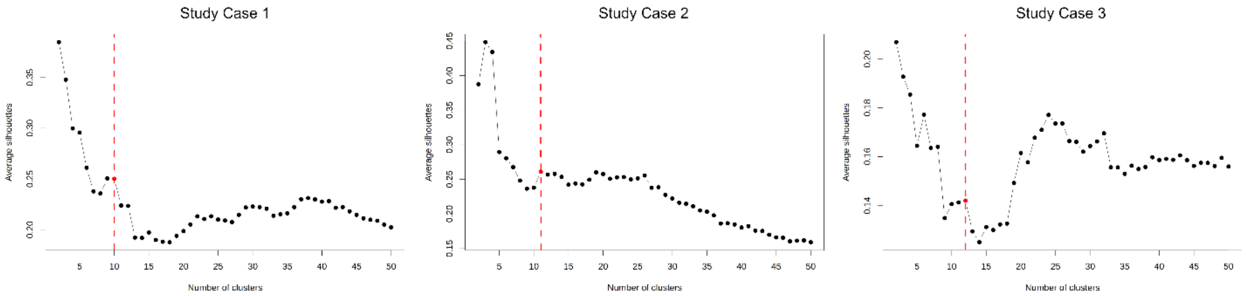

Figure S3: Silhouette profiles for the three study cases. The optimal number of clusters (red) was chosen as the one with the highest silhouette score in the range 9-15.

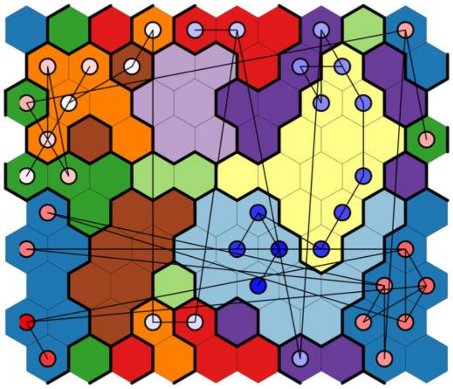

Figure S4: An example of pathways traced on a SOM trained using periodic boundary condition.

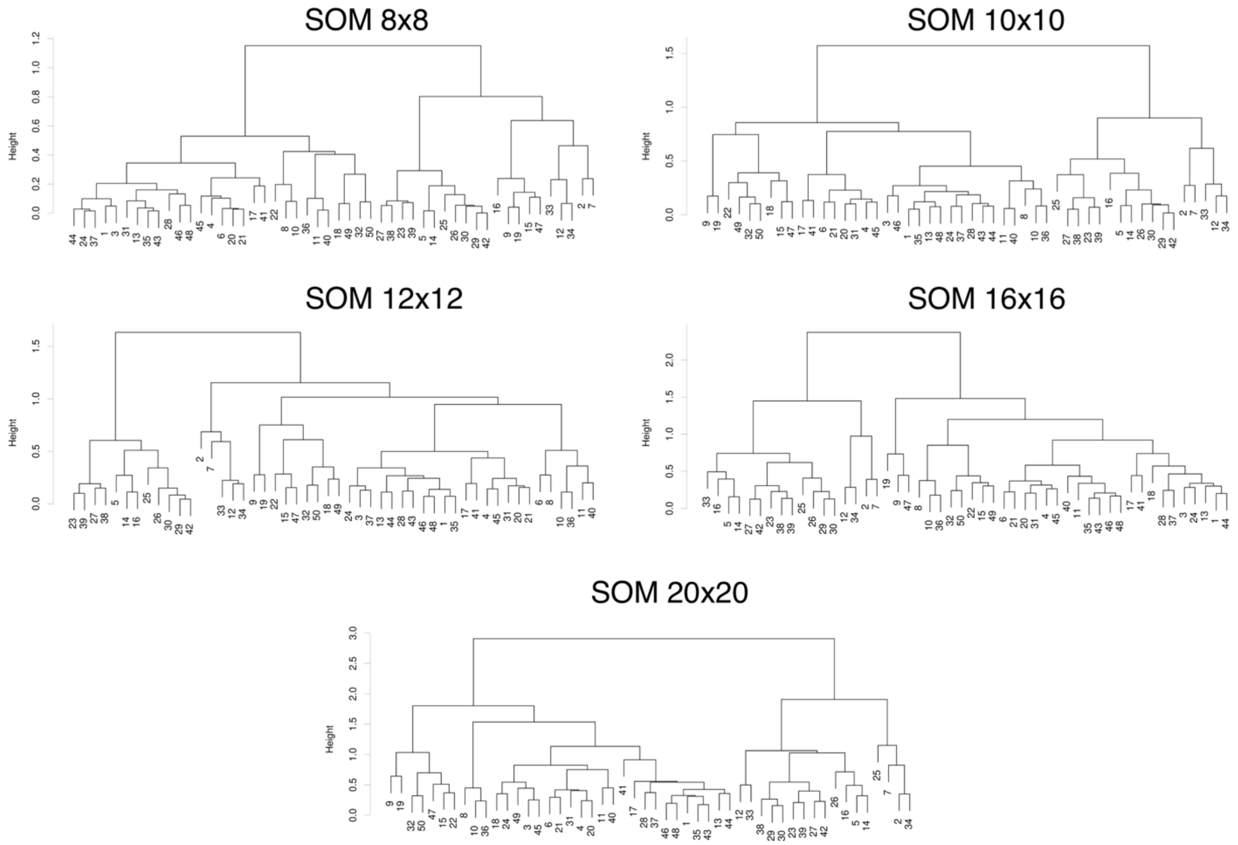

Figure S5: Dendrograms of hierarchical clustering of the pathways for the study-case 1 obtained using different SOM dimensions.

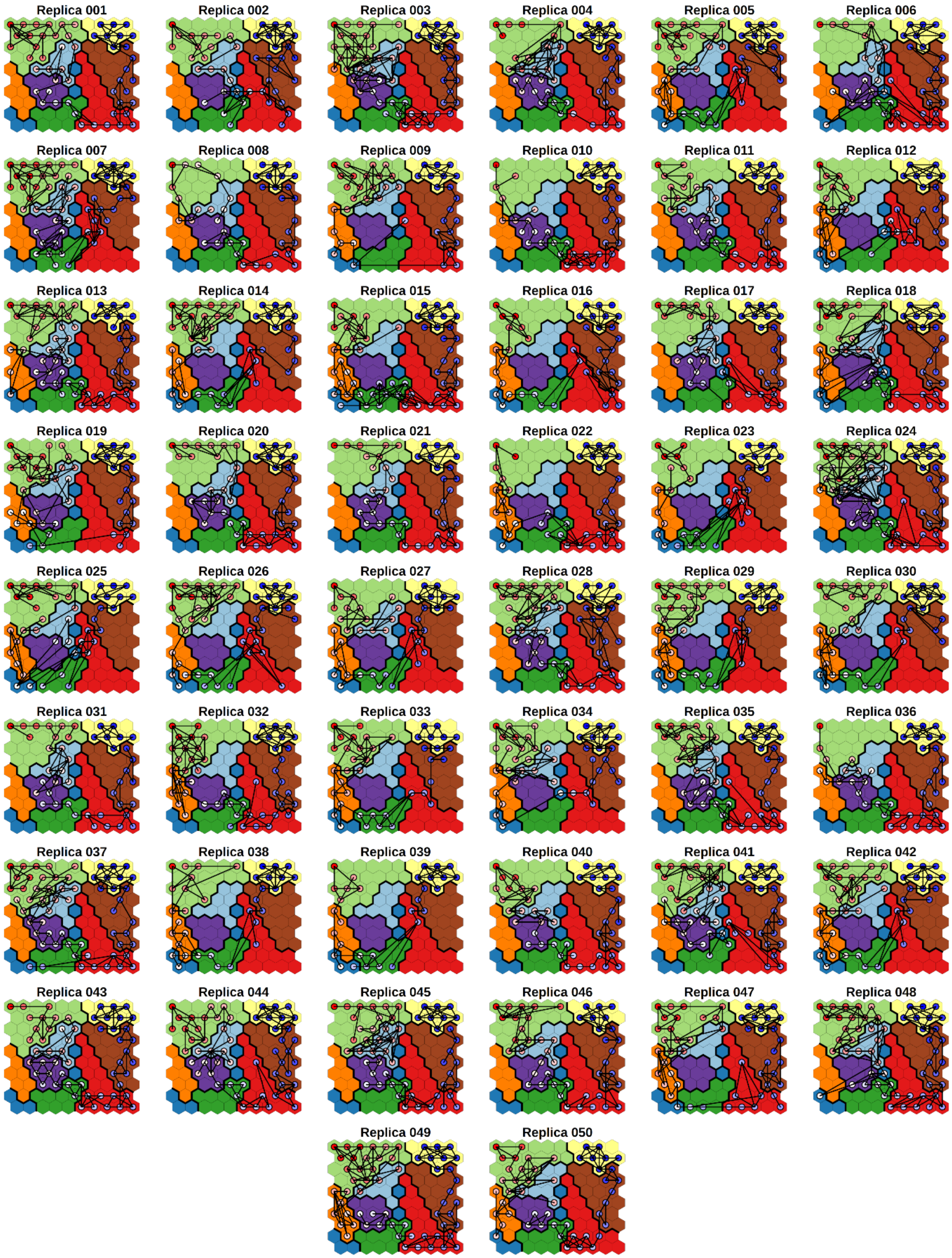

Figure S6: Tracing of the pathways on the trained SOM for the SMD replicas of THS-020 unbinding from HIF-2α (study case 1).

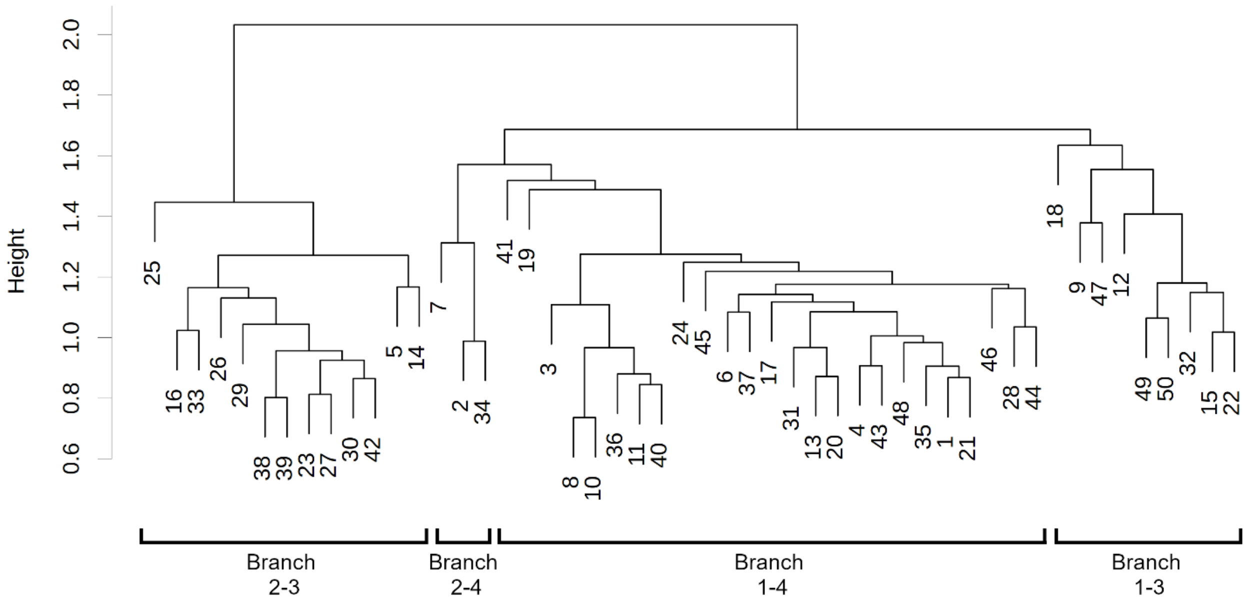

Figure S7: Dendrogram of hierarchical clustering of the pathways followed by different replicas for the study case 1 (HIF-2α SMD simulations).

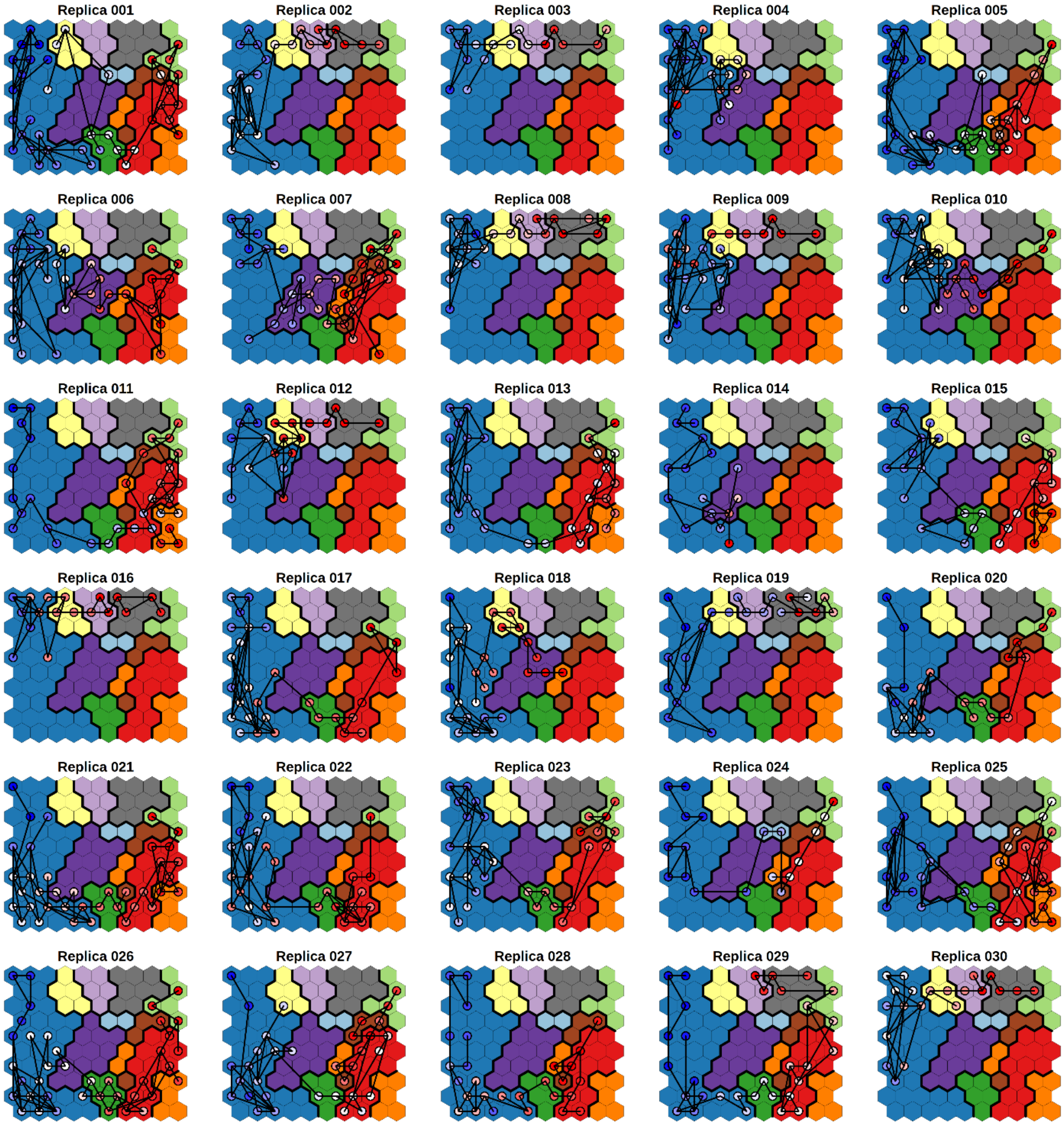

Figure S8: Tracing of the pathways on the trained SOM for the MetaD replicas of GC7 unbinding from DHS (study case 2).

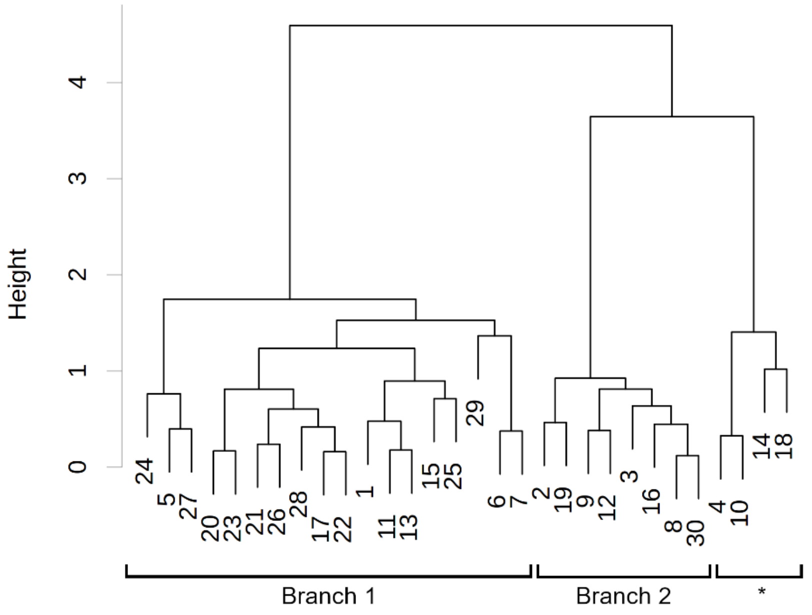

Figure S9: Dendrogram of hierarchical clustering of the pathways followed by different replicas for the study case 2 (DHS MetaD simulations). Replicas can be assigned to branch 1 or branch 2 of the network, in good agreement with the clustering, except for four replicas indicated with (*) in the dendrograms. Most of these replicas did not reach a completely unbound state.

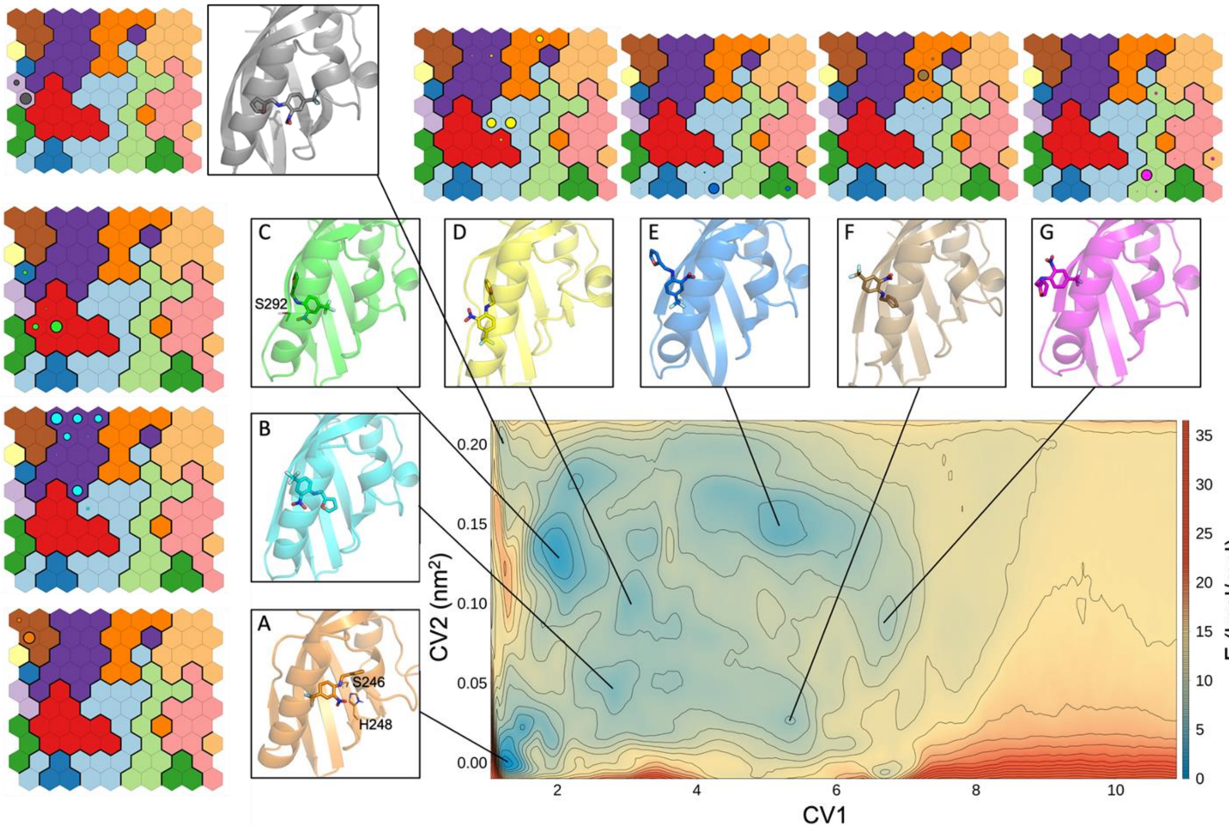

Figure S10: Mapping of frames belonging to each free-energy basin on the SOM for the study-case 3. Letters in squares correspond to the free energy states identified in the original paper.[4] Cluster I (top left, grey) do not correspond to any of the original lowest free-energy states of the map. Figure adapted from Fig 4. in Ref [4].

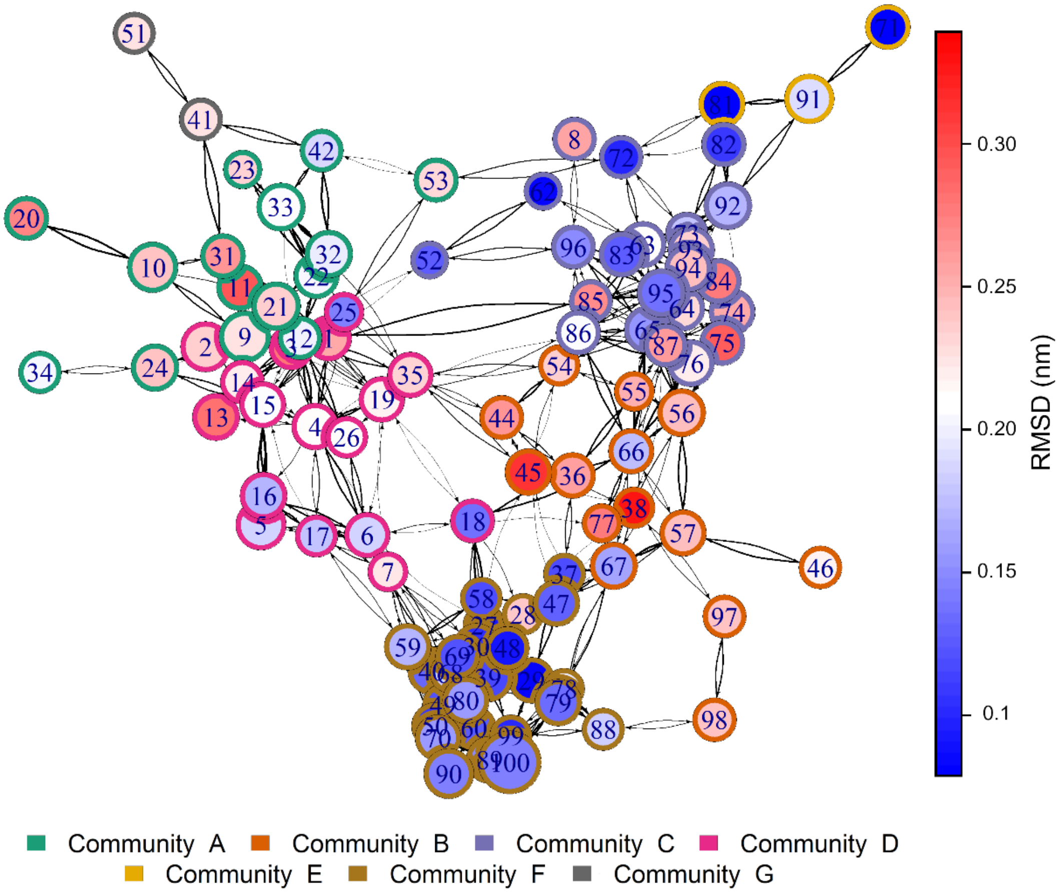

Figure S11: Transition network for the MetaD simulation of THS-020 binding to HIF-2α. Nodes are colored according to the average RMSD value of residues belonging to the mouth of the cavity (computed on backbone atoms of residues 288-293 and residues 302-306). The circle around each node is colored according to the community it belongs to.

REFERENCES

(1) Sokal, R. R.; Rohlf, F. J. The comparison of dendrograms by objective methods **1962**, *11* (2), 33–40. https://doi.org/10.2307/1217208.

(2) Baker, F. B. Stability of Two Hierarchical Grouping Techniques Case 1: Sensitivity to Data Errors. *Journal of the American Statistical Association* **1974**, *69* (346), 440. https://doi.org/10.2307/2285675.

(3) Galili, T. Dendextend: An R Package for Visualizing, Adjusting and Comparing Trees of Hierarchical Clustering. *Bioinformatics* **2015**, *31* (22), 3718–3720. https://doi.org/10.1093/bioinformatics/btv428.

(4) Callea, L.; Bonati, L.; Motta, S. Metadynamics-Based Approaches for Modeling the Hypoxia-Inducible Factor 2α Ligand Binding Process. *Journal of Chemical Theory and Computation* **2021**, *18*. https://doi.org/10.1021/acs.jctc.1c00114.
